# Posterior sagittal approach in complicated Swenson's pull-through

**DOI:** 10.4103/0971-9261.42567

**Published:** 2008

**Authors:** O. A. Sowande, O. Adejuyigbe

**Affiliations:** Department of Surgery, Pediatric Surgery Unit, Obafemi Awolowo University Teaching Hospital, PMB 5538, Ile Ife, Osun State, Nigeria

**Keywords:** Complications, posterior sagittal approach, Swenson pull-through

## Abstract

**Context:**

Swenson's pull-through is one of the standard operations for the treatment of children with Hirschsprung's disease. Complications arising from the operation are difficult to treat because of fibrosis in the pelvis. The posterior sagittal approach may be a safer alternative.

**Aims:**

The aim of this paper is to highlight our experience with the use of the posterior sagittal trans-sphincteric approach to treat unusual complications of Swenson's pull-through.

**Settings and Design:**

A retrospective study of four patients who had posterior sagittal repair of their complications of Swenson pull-through at the Obafemi Awolowo University Teaching Hospital, Ile Ife, Nigeria.

**Materials and Methods:**

Four cases of Hirschsprung's disease that developed post-Swenson pull-through complications are presented. There were three males and one female. Their age ranged between 10 months and 15 years. The patients had rectovaginal fistula, rectourethral fistula, high trans-sphincteric fistula-in-ano and complete anastomotic disruption.

**Result:**

All the patients were successfully treated using the posterior sagittal approach. The approach was used twice in one patient without significant sequelae. The three patients were old enough to be assessed and had a Kelly score of 4-6 at follow-up.

**Conclusion:**

The posterior sagittal technique offers a safe approach to treat the complications of Swenson pull-through.

## INTRODUCTION

Swenson's operation is one of the standard operations for the treatment of children with Hirschsprung's disease. However, in up to 6% of the patients, major surgical interventions or even a redo operation is necessary for the treatment of associated complications.[1–3] Very often these redo operations are carried out by abdominoperineal approach, which can be very difficult because of adhesions increasing the risk of further injury to adjacent structures. The posterior sagittal anorectoplasty approach was popularized by Pena and deVries for the treatment of anorectal malformations in children, but it has found several new applications in the management of anorectal conditions both in adults and children alike. There are reports of the application of the technique in the primary treatment of Hirschsprung's disease and also in the repair of postoperative complications of standard pull-through techniques.[4–7] This paper reviewed four cases of uncommon complications of Swenson's operation for Hirschsprung's disease treated with the posterior sagittal technique and their functional outcome.

## MATERIALS AND METHODS

Four patients who had posterior sagittal repair of complicated Swenson pull-through were included in this study. All the patients had preoperative bowel preparation and were on colostomy before the secondary surgery. Continence was assessed using the Kelly score.[8]

## CASE REPORTS

### Case 1: Swenson's pull-through complicated by complete anastomotic disruption

A 10-month-old infant with histologically confirmed Hirschsprung's disease had a primary Swenson pull-through with tube cecostomy for fecal diversion. He developed postoperative intestinal obstruction which necessitated laparotomy. At laparotomy, the colon proximal to the anastomosis was dilated. While attempting to mobilize the colon for a colostomy, there was a complete anastomotic disruption in the pelvis. The dilated colon was excised leaving only around 15 cm of colon to be available. The distal end of the colon was closed and fixed in the retrorectal space, and the laparotomy wound closed. The child was repositioned in the knee-elbow position, and through a posterior sagittal incision extending from the sacrum to the anus, the striated muscle complex was divided strictly in the midline. The colon was freed and pulled down into the wound and circumferentially anastomosed to the rectal stump using 4/0 vicryl sutures. He started passing feces spontaneously per rectum, but by the fifth day, there was complete disruption again with retraction of the bowel and breakdown of the sagittal wound. Prompt laparotomy was done and the end of the colon was fashioned into an end colostomy in the right iliac fossa. The perianal wound was healed with sitz bath and dressings. After 8 months, a repeat laparotomy was done, end stoma was mobilized from the right iliac fossa, presacral space was developed and colon fixed in the pelvis at the level of the coccyx. The laparotomy wound was closed. Through a repeat posterior sagittal incision, the rectal stump that had been stenosed was opened, mucosa was excised and colon was mobilized and re-anastomosed to the residual anal stump. Postoperatively, there was a small colocutaneous fistula in the sagittal wound that healed spontaneously. After 6 months of follow-up, he passed stool spontaneously, but up to 8 times a day. He was apparently fully continent of feces.

### Case 2: Swenson's pull-through complicated by rectovaginal fistula

A 15-month-old female with Hirschsprung's disease referred to our unit with a left sigmoid loop colostomy. She had Swenson's rectosigmoidectomy with a cecostomy tube cover. She had immediate postoperative vaginal bleeding followed by fecal discharge per vagina 5 days postoperatively. A diagnosis of the rectovaginal fistula was made. The cecostomy tube was removed and a descending loop colostomy was fashioned. The fistula failed to heal after 3 months, following which examination under anaesthesia (EUA) and dye test via the distal stoma was performed. A fistula with a diameter of 1 cm was found in the posterior vagina wall. An attempt was made to repair the fistula through the anterior perineal/trans-vaginal approach; however, this also broke down. Two months later, she had another repair via the posterior sagittal route. Through the posterior sagittal approach, the rectum was opened and the fistula was identified. A circumferential incision around the fistula was made and this was freed from the rectum. The fistula was then closed in 2 layers using 4/0 PDS suture. The opening in the anterior and posterior wall of the rectum was also closed in two layers. The sphincter muscles were then repaired and the wound closed. The colostomy was closed 6-weeks later when the perineal wound was thought to have healed. She developed rectocutaneous fistula through the sagittal incision that healed with conservative management. At more than 3 years of follow-up, she showed a Kelly score of 4 as she had occasional accidents at night, while being fully continent during the day.

### Case 3: Swenson's pull-through complicated by rectourethral fistula

A 11-year-old male patient who had rectal biopsy confirmed Hirschsprung's disease 8½ years earlier, underwent a one-stage Swenson's operation performed with tube cecostomy for fecal diversion. He started passing stool spontaneously per rectum by the third day. EUA at day 10 revealed a posterior anastomotic dehiscence, which was managed conservatively. He was readmitted after 4 weeks with straining at defecation; on EUA, it was found to be due to a tight anastomotic stricture barely accepting the tip of the fingers. Sequential Hegar's dilatation was started; however, a forceful attempt during one of the sessions was followed by severe bleeding per rectum. One week later, he started passing feces per urethra due to a rectourethral fistula. The rectourethral fistula was confirmed by barium enema. A right transverse divided colostomy was fashioned, but the fistula failed to heal. Six months after the initial pull-through, he was approached with a posterior sagittal trans-rectal approach, which was similar to the patient in case 2. The fistula was freed from the rectum and closed in two layers over an indwelling urethral catheter. On the postoperative second day, the urethral catheter got dislodged. He started leaking urine via the distal colostomy stoma, suggesting a recurrence of the fistula. A distal stoma colostogram confirmed the recurrence. He remained on colostomy for 6 months without the healing of the fistula. A repeat attempt was made to close the fistula through the posterior sagittal approach. The rectum was divided at approximately 3 cm from the dentate line. The mucosa of the distal rectum was removed and the rectum was placed inside the distal rectal muscle cuff and anastomosed above the dentate line. The fistula was bypassed completely. The posterior cuff of the rectum was closed in two layers, and the sphincter muscle was reconstructed. The urinary leak stopped spontaneously and the colostomy was closed 6 weeks later. The child did well postoperatively. Six years later he is fully continent of flatus and feces with a Kelly score of 6.

### Case 4: Swenson's pull-through complicated by high trans-sphincteric fistula-in-ano and retrorectal abscess

A 15-year-old male was admitted with constipation and gross abdominal distension since birth. Barium enema and rectal biopsy confirmed Hirschsprung's disease. He had a primary Swenson's pull-through, which was complicated by anastomotic dehiscence, peritonitis and retrorectal abscess and multiple perianal fistulae. He had emergency laparotomy, peritoneal lavage and right transverse colostomy with frequent rectal irrigation. The anastomotic dehiscence failed to heal, and he continued to have recurrent per anal purulent discharge associated with a persistent fistula-in-ano; which was suggestive of a high trans-sphincteric fistula communicating with the peri-rectal abscess cavity on fistulogram. Two years following the initial pull-through, he had a posterior sagittal repair, through which the fistula tract was excised and the abscess cavity was obliterated. During the surgery, all the layers including the sphincteric muscle were divided, the retrorectal abscess drained and the cavity was irrigated with hydrogen peroxide and saline. The fistula tract was excised. The rectum was mobilized and re-anastomosed to the anal stump under direct vision. The sphincteric muscle was reconstructed and the sagittal wound was closed in layers. The wound healed satisfactorily, and the colostomy was closed 3 weeks later. After 8 years of follow-up, he was fully continent of feces and flatus with a Kelly score of 6.

## RESULTS

The demographic characteristics, indications, complications and follow-up outcomes of the patients are outlined in Table 1. All the patients were successfully treated using the posterior sagittal approach. The approach was used twice in one patient without significant sequelae. All the patients are continent with a Kelly score ranging from 4 to 6. Follow up ranged between 6 months to 8 years.

**Table 1:** Showing the indications and outcome of the posterior sagittal repair in the four patients

Age of the patient	Type of primary surgery	Complication(s) of primary pullthrough	Secondary surgery	Postoperative complications	Continence	Follow up
10 months	Primary Swenson PT with tube cecostomy	Postoperatve intestinal obstruction with complete anastomotic dehiscence requiring terminal colostomy	Posterior sagittal transsphincteric repair	Colocutaneous fistula (healed spontaneously)	Up to 8 motions per day but continent	6 months
15 months	Swenson PT with tube cecostomy	Rectovaginal fistula	Posterior sagittal transrectal repair	Rectocutaneous fistula (healed spontaneously)	Occasional accidents at night	3 years
**11 years	Primary Swenson PT with tube cecostomy	Anastomotic Disruption with Rectourethral fistula	Posterior sagittal transsphincteric repair	Nil	Fully continent	6 years
15 years	Primary Swenson PT	High trans-sphincteric fistula with retrorectal abscess	Posterior sagittal transsphincteric repair	Nil	Fully continent	8 years

** This patient had the procedure twice after the recurrence of the fistula

## DISCUSSION

While performing the Swenson's pull-through for Hirschsprung's disease, the abdominal mobilization of the aganglionic segment down to the dentate line may be hazardous and can be complicated by injuries to other adjacent structures. Even in the absence of such injuries, the procedure itself can be complicated by anastomotic dehiscence, stricture/stenosis and abscess formations and occasionally by rectourinary or rectovaginal fistula.[2] The secondary re-operation following Swenson's pull-through operations is estimated to be around 6%.[2,3] Surgical reconstruction following complications such as rectourinary fistula and rectovaginal fistula or anastomotic complications can be difficult. This is because of the fibrous adhesions between the rectum and the surrounding structures, including the fistulae as well as with the pelvic organs, particulary on following Swenson's type pull-through. In these instances, the endoanal pull-through and the Duhamel operations are alternatives; however, they also involve difficult pelvic dissection. In all the patients presented, we have been able to effectively repair and reconstruct using the posterior sagittal approach. This approach was designed for the treatment of anorectal malformations in children by Pena and deVries in 1982. Since its introduction, it has found a variety of other clinical applications in the treatment of congenital and acquired disorders in adults and children likewise.[4,9,10] Recently, the posterior sagittal approach has been applied in the primary treatment of rectosigmoid Hirschsprung's disease with successful outcome.[5,6] The technique has also been applied in the endorectal repair of rectovaginal and rectourethral fistulae in children following previous pull-through.[3] We have applied the posterior sagittal approach to treat a variety of unusual complications of Swenson's operation, namely, rectovaginal fistula, rectourethral fistula, complete anastomotic dehiscence and high trans-sphincteric fistula-in-ano with retrorectal abscess and anastomotic stricture. The approach has been used with satisfactory functional outcomes. The posterior sagittal approach offers the advantage of visualization of the fistula and easy repair of the fistula tract, while it also allows direct anastomosis of the proximal colon to the anal stump in a manner similar to that described by Aggarwal *et al.*[11] The reconstruction of the sphincteric muscles under direct vision was done in all the patients without problems and all are fully continent, except for patient no. 1, who has occasional accidents at night. It should also be noted that two of our patients had division of the sphincteric muscles twice without any deleterious effect. These confirmed the experimental observation that division of the sphincteric muscle is not associated with any deleterious effect,[12] provided that there is strict adherence to the midline in order to preserve the vascular supply and innervations of the sphincteric muscles. Strictly adhering to this basic tenet of this surgery one can avoid complications and possibly the operation can be repeated if necessary, as in our first and third patients. There was no need to divide the fistula again in the second patient because of the dense adhesions between the urethra and the rectum, but the fistula was bypassed completely without further recurrence. This confirmed the observation of Kubota *et al.*[7] that division of the fistula or interposition of various vascularized tissue between the rectum and the fistula may not be necessary at all when this approach is used.

With these observations in the presented patients we conclude that posterior sagittal approach effectively deals with post swenson's pull through procedure complications. Anorectal continence is always preserved as the reconstruction of sphincteric muscles is done under vision.
